#  Response to Wang and Luo

**DOI:** 10.1186/1741-7007-10-32

**Published:** 2012-04-18

**Authors:** Marc Rehmsmeier

**Affiliations:** 1 Computational Biology Unit, Uni Computing, Uni Research, Høyteknologisenteret, Thormøhlensgate 55, 5008 Bergen, Norway

## Abstract

This article is a response to Wang and Luo.

See correspondence article http://www.biomedcentral.com/1741-7007/10/30 and the original research article http://www.biomedcentral.com/1741-7007/9/24.

## Background

As stated in Matzke *et al. *[1], '[c]haracterizing the ways in which polyploid genomes evolve is essential for an understanding of plant and vertebrate evolution'. In our recent study [2], we investigated whether polyploidy has an influence on the frequency of meiotic recombination. In a seed fluorescence assay we observed increased meiotic recombination frequencies (MRFs) between two transgenic marker loci in autotetraploids, when compared to diploids. Results from newly synthesized allotetraploids corroborated our findings, indicating that the observed increase of MRF in tetraploids is independent of the formation of multivalents [2]. Wang and Luo argue in their correspondence that the analysis of our marker data needs to be formulated on the basis of disomic and tetrasomic inheritance models and apply a previously developed method [3].

### Maximum likelihood estimation of parameters

Wang and Luo argue that our calculation 'did not use full information of the data. For example, the individuals with yellow seeds were not taken into consideration when counting for recombination events.' The solution of *r* that we used in the selfing case is the one root in [0,1] of the equation 2*r *- *r*^2 ^= 2(*n*_2 _+ *n*_3_)/*n *(in Wang and Luo's notation), namely $\hat{r}=1-\sqrt{1-2\left(n_{2}+n_{3}\right)/n}$ which is indeed in general not the maximum likelihood solution but the simple formula from [4]. Nevertheless, the resulting estimates are nearly identical to Wang and Luo's estimates (see below). Furthermore, the formula we used in the backcross cases, $\hat{r}=\left(n_{2}+n_{3}\right)/n$, is a maximum likelihood solution (that of the equation system *n*_1 _= *n*(1 - *r*)/2, *n*_2 _= *nr*/2, *n*_3 _= *nr*/2, *n*_4 _= *n*(1 - *r*)/2; details not shown).

### Marker location

Wang and Luo state that the green marker 'is nearer to the centromere than the red marker locus'. However, it is the red marker that is nearer to the centromere, as stated in our original article ('Both inserts are located on the top arm of chromosome 3, GFP distal and RFP proximal' [2]). Should Wang and Luo indeed have modeled the locations of the markers incorrectly, it would in principle be difficult to interpret the resulting estimates. However, this response also includes the author's own calculations on the basis of the model from [3], with correct marker locations, and the resulting estimates are very similar. This suggests that the overall estimate of MRF is not very sensitive to these aspects.

### Applying Luo *et al*.'s model

Wang and Luo apply the quadrivalent model from [3] to the seed fluorescence assay in [2] and derive a set of equations that express the probabilities of the various fluorescences (green-only, red-only, both/yellow, none/brown) in terms of MRF *r *and coefficient of double reduction *α*. Details on how these equations were derived are not given, but the author of this response arrives at a different set of equations with the following procedure: For each of the 11 modes of gamete formation in Table 1 in [3] and for each gamete genotype (carrying one or the other marker, both markers, or none) it was determined how many of the gametes that constitute that mode (column 1 in Table 1 in [3]) are consistent with that genotype. This number, divided by the overall number of gametes that constitute the mode (column 2 in Table 1 in [3]), and multiplied by the probability of the mode (column 4 in Table 1 in [3]) is the probability of the mode when conditioned on the genotype. All such probabilities summed up for all gamete modes is the probability of the gamete genotype in terms of *r *and *α*. The final gamete probabilities, after simplification, are:

$$\begin{array}{c} p_{\text{green}-\text{only}}\left(r,\alpha \right)=-\left(2+\alpha \right)\left(-6+r\right)r/36\\ p_{\text{red}-\text{only}}\left(r,\alpha \right)=r\left(6-2r+\alpha \left(-6+5r\right)\right)/12\\ p_{\text{both}}\left(r,\alpha \right)=\left(\alpha \left(-3+6r-5r^{2}\right)+2\left(3-3r+r^{2}\right)\right)/12\\ p_{\text{none}}\left(r,\alpha \right)=\left(2+\alpha \right){\left(-3+r\right)}^{2}/36 \end{array}$$

which overall do not correspond to Wang and Luo's *g*_1 _to *g*_4 _(except for *p*_both _which is equal to *g*_1_). For the calculations below, the gamete genotype probabilities were combined into seed phenotype probabilities in agreement with Wang and Luo's definition of their *f*_1 _to *f*_4_.

### Complete quadrivalent pairing

Wang and Luo assume complete quadrivalent pairing, that is they do not allow for a mixture of quadrivalents and bivalents as has been done in [3], there governed by a mixing parameter *λ*. Not only would it be interesting to see what estimates of *λ *one would obtain on the data from [2], but considerable frequencies of bivalents both in established and in newly formed autotetraploid *Arabidopsis thaliana *have been reported [5]. One could imagine that a mixture model would result in MRF estimates that are closer to the ones reported in [2].

### Double reduction

Wang and Luo argue that the presence of significant double reduction at the markers emphasizes the necessity of taking the tetrasomic nature of autotetraploid meiosis into account. However, the qualitative similarity of estimated MRFs from our simple bivalent model and Wang and Luo's full quadrivalent model, when compared to MRF estimates in diploids, questions the validity of this statement to some extent. In addition, the author's own calculations result in an estimate of the coefficient of double reduction *α *of 0.014 with a standard deviation of 0.018, as determined from estimates across the biological replicates (see further below for a discussion of this aspect), which is considerably smaller than the *α *of 0.0676 as reported by Wang and Luo. Also, as discussed above, a mixture of quadrivalent and bivalent pairing might further weaken this argument.

### Self-pollination versus backcross experiments

In the selfing cases, estimated recombination frequencies are mixtures of female and male recombination frequencies, both in Wang and Luo's and in our treatment, with the exact nature of this mixture not being discernible from the experiments performed. While these mixed selfing MRFs are indicative of the overall differences we observe between diploids and tetraploids, the sex-specific backcross experiments might be more meaningful. Pecinka *et al. *report significant differences in MRF also in these cases, and it would be interesting to see what Wang and Luo would calculate with their approach.

### Biological replicates

Wang and Luo estimate MRFs from the overall sums of seed counts. In [2], MRFs in Table 1 were calculated from overall sums of seed counts, and MRFs in the supporting material were calculated from overall sums of seed counts and also from biological replicates individually. Standard deviations were calculated across biological replicates and thus represent biological variance, in contrast to the asymptotic variance of the maximum likelihood estimator as used by Wang and Luo.

### Differences in estimated recombination frequencies

Wang and Luo compare their diploid selfing MRF estimate of 0.1643 with our estimate of 0.154 [2]. It has now become apparent that in the original article the backcross formula was erroneously applied, instead of the correct selfing formula from [4]. With the correct formula the result is 0.168. Calculating individually on the three replicates (additional file 1 in [2]) gives an average estimate of 0.174. Using the maximum likelihood estimator, as suggested by Wang and Luo, but again individually on the three replicates, gives an average estimate of 0.176. In summary, upon correction of the formula used and comparing like calculations, the results of Wang and Luo's formula and our formula are nearly identical.

In the allotetraploid selfing case, Wang and Luo compare their estimate of 0.2770 with ours of 0.241 [2]. Again, in the original article the backcross formula was applied and not the selfing formula. With the correct formula the result is 0.280. Applying the correct formula individually on the five replicates (additional file 3 in [2]) results in an average estimate of 0.276, and using the maximum likelihood estimator individually on the five replicates results in an average estimate of 0.273. Again, in summary, upon correction of the formula used and comparing like calculations, the results from the maximum likelihood estimator suggested by Wang and Luo and the simple one of [4] are nearly identical.

In the autotetraploid selfing case, Wang and Luo compare their estimate of 0.3048 with ours of 0.205. Again, in the original article the incorrect backcross formula was applied and not the selfing formula. With the correct formula the result is 0.232. Applying again the simple formula individually to the 10 biological replicates (additional file 2 in [2]) gives an average estimate of 0.231. Using the maximum likelihood estimator individually on the biological replicates results in an average estimate of 0.227, and used on the overall sums of seed counts results in an estimate of 0.228. Using the tetraploid model as derived from [3] with correct marker location and on the individual crosses results in an average estimate of 0.290, and on the overall sums of seed counts results in an estimate of 0.293. In summary, the results from a model of tetraploid meiosis that takes quadrivalent formation into account more strongly emphasize the differences between diploid and autotetraploid selfing MRFs. However, the discrepancy in differences, when calculated correctly and in comparable ways, is not as large as portrayed by Wang and Luo and if anything only strengthens our original findings. Also note that with Wang and Luo's model (both in their correspondence and in this response) complete quadrivalent pairing was assumed and that upon using a mixed quadrivalent-bivalent model the differences in results might be further reduced. It is compelling though how close the autotetraploid MRF is to the allotetraploid MRF when calculated with the quadrivalent model, as opposed to the simple bivalent model, indicating that autotetraploid meiosis is indeed more accurately represented by a model that takes double reduction into account.

The above results are summarized in Table 1 together with standard deviations where applicable.

**Table 1:** Meiotic recombination frequencies from different calculations

Ploidy (meiosis)	Simple^a ^averaged^b^	ML^c ^averaged	Simple overall^d^	ML overall	Luo *et al*.^e ^averaged	Luo *et al. *overall
Diploid (selfing)	0.174 ± 0.011	0.176 ± 0.023	0.168	0.164	NA	NA
Allotetraploid (selfing)	0.276 ± 0.026	0.273 ± 0.031	0.280	0.277	NA	NA
Autotetraploid (selfing)	0.231 ± 0.014	0.227 ± 0.012	0.232	0.228	0.290 ± 0.017	0.293

All values calculated from data in [2], supporting material.

^a^Simple: diploid formula from [4].

^b^Averaged: average of estimates across biological replicates, ± standard deviation of estimates.

^c^ML: maximum likelihood estimator, as suggested by Wang and Luo.

^d^Overall: estimate on overall seed counts.

^e^Complete quadrivalent model as derived from [3], with correct marker locations.

Wang and Luo also argue that their study differs from ours in the inferred order in MRF of allotetraploids and autotetraploids. However, a comparison of allotetraploids and autotetraploids was not the goal of our study. Our goal was a comparison of diploids and tetraploids, may the latter be allo- or autotetraploids. The main result of our study is that tetraploids show significantly larger MRFs between the two loci studied in comparison to diploids. While the order of tetraploid frequencies was of no importance to us, the fact that the differences between allo- and autotetraploids are small when compared to the differences between diploids and tetraploids shows the robustness of our main result.

## Conclusions

During our work, which was ultimately published in [2], the author of this response developed a mathematical model of tetraploid meiosis that also included the formation of quadrivalents. From simulations it was concluded that the number of seeds available was too small to robustly estimate coefficients of double reduction (data not shown). MRF between the two loci, however, could very robustly be estimated, and these estimates were close to estimates from a bivalent-only model, the model in the end used in [2], also for autotetraploids (data not shown). In fact, setting the probability of quadrivalent formation to zero in a mixed bivalent-quadrivalent tetraploid model, reduces this model to the diploid formulas.

The author of this response shares the opinion that proper models of tetraploid meiosis, such as the one from [3], have important merits, such as their ability to calculate coefficients of double reduction and higher accuracy in estimating recombination frequencies, which is of great importance in mapping endeavors and in general highly relevant under evolutionary aspects (see for example [3] and references therein). However, in the context of the work published in [2] - a comparison of diploid and tetraploid meiotic recombination frequencies - these models, while making estimates more consistent, do not challenge the conclusions drawn from a simplified model. This of course can only be determined by applying the various models in the first place, and Wang and Luo are to be thanked for bringing this interesting topic to the attention of readers.

## Abbreviations

MRF: meiotic recombination frequency.
